# Pace of adoption of alternatives to animal-source foods is an important factor in reaching climate goals

**DOI:** 10.1038/s41598-025-07866-x

**Published:** 2025-07-02

**Authors:** Galina Hale, Vlad Oncescu, Ritesh Bhangale

**Affiliations:** 1 University of California Santa Cruz, NBER, CEPR, Santa Cruz, USA; 2 Accenture, New York, USA

**Keywords:** Climate change, Food system sustainability, Dietary change, Alternative proteins, Plant-based products, Cultured products, Climate-change mitigation, Climate-change mitigation, Sustainability

## Abstract

The global food system is a significant contributor to greenhouse gas emissions that drive climate change. Animal agriculture accounts for a large share of food-system emissions, both directly and through the production of animal feed. Global population growth and rising incomes imply a further increase in demand for animal-source foods if current trends persist. Limiting global warming to the targets set by the international community will not be possible without the rapid reduction of a substantial share of animal-source foods. We show that the rapid adoption of alternatives to animal-source foods, such as plant-only diets or plant-based, cultured, or fermentation-derived analogs to animal products, can be consistent with climate goals while satisfying global demand for calories and protein. Importantly, timing is crucial: the longer the delay in adopting alternatives, the larger the share of the diet that must shift away from animal-source food by 2050 for the food system to remain within its carbon budget.

The world economy is not on track to reach climate goals^1^. In recent years, it has become evident that constraining global temperature increase will require not only changes in energy production and transportation, but also changes in the food system. Food systems are responsible for as much as a third of global anthropogenic greenhouse gas (GHG) emissions^2^. Therefore, the Paris Agreement climate goals cannot be achieved without changing the food system^3,4^. Because of the disproportionate GHG emissions from the production of animal-source foods, relative to their overall contribution to global caloric intake, a reduction in reliance on animal-source food can drastically reduce food system GHG emissions^5–8^.

A global switch to plant-based diets is a potential solution, but resistance to reducing consumption of animal-source foods (ASF) is well documented and an upward trend in global consumption of ASF continues^9,10^. Thus, it is unlikely that a sufficient share of consumers will shift to plant-only diets fast enough to achieve climate goals, and therefore there is a role for alternatives to ASF that rely on new technologies to make products more acceptable to consumers than most products in the market today. These technologies, while not emission-free, have the potential for substantially lower emissions than ASF, especially given the projected energy transition to clean power.

Research has emphasized the need to increase the share of plants and reduce the share of animal-source products in the average diet to achieve climate goals and preserve biodiversity^11–13^. Eliminating greenhouse gas emissions from livestock and allowing native ecosystems to regrow on the land currently used to feed livestock would be equivalent to a 68% reduction in carbon dioxide emissions^14^. Replacing just 50% of major animal products with alternatives by 2050 is estimated to reduce land use GHG emissions by 31% compared to 2020^15^. These alternatives, however, also have a GHG footprint. While dietary changes are a partial solution^16^, such changes tend to be slow without the introduction of alternatives that are attractive to consumers in terms of price, taste, texture, nutritional value, and other attributes that consumers value^17^.

We contribute to this growing literature by providing a dynamic quantitative analysis of the global cumulative GHG emissions from the food industry under different dietary scenarios, taking into account possible realistic consumer adoption paths for dietary changes. In particular, we model continuing trends in population growth, caloric intake, and dietary preferences by region, and calculate the GHG emissions from the food sector that are consistent with producing enough calories (or enough protein) to satisfy the resulting demand. Our results reveal that the dynamics of the transition to a sustainable food system are key to achieving climate goals: the two crucial parameters are the timing of the transition take-off and the ultimate share of ASF replaced, while the specific pace of the transition following take-off is less important quantitatively.

We group alternatives to animal-source food products into two categories: plant-based, which are available to consumers and are already increasing their market share^18^ and “cultured,” which includes new technologies for protein production such as cellular agriculture (or cultivated alternatives), precision fermentation, biomass fermentation, molecular farming, and potentially other innovations, which are with few exceptions, still mostly in the development stage or awaiting regulatory approvals. Production of these alternatives, especially when powered by clean energy, has a much lower GHG footprint than ASF^19–22^. We consider scenarios in which ASF are replaced by such alternatives to evaluate the potential for this transition to lead to a sustainable food system when compared to business-as-usual, plant-only diet, and healthy diet scenarios that do not include the introduction of these alternatives to ASF.

Our approach to the dynamics of food system sustainability transition is distinct from previous studies in that we model the transition based on consumer acceptance models rather than pathways derived from emission reduction needs from climate scenarios. Thus, we provide calculations of cumulative emissions between 2020 and 2050 under conventional assumptions about the dynamics of consumer acceptance of new products. We find that compared to the business-as-usual (BAU) scenario in which most global diets converge toward a Western diet by 2050^23^, a substantial reduction in cumulative food sector GHG emissions can be achieved with shifts to alternatives to ASF or a plant-only diet. The emission reduction crucially depends on the starting point of the transition: the longer the delay before the transition begins, the larger the share of ASF that needs to be replaced by 2050 for the same target cumulative emissions.

Our analysis provides sufficient information for the range of estimates of the food system carbon budget between 2020 and 2050, the pace of transition to sustainability, and other parameters. However, we use a benchmark set of parameters to provide specific numbers for our quantitative results. Our BAU projections lead to an estimated cumulative emissions from the food system between 2020 and 2050 of 607 GtCO2e if diets converge to 3220 kcal per person (current global average), or 639 GtCO2 if diets converge to 3515 kcal per person (current developed-countries average), with diet change contributing 12.6%, population growth 13%, and caloric intake changes 4.7% to this total. If the goal is to keep global temperature rise under 1.5 degrees C with a 67% probability, IPCC estimates the remaining carbon budget at 1170 GtCO2e. Assuming the food sector continues contributing about a third of global GHG emissions^2^, this leaves a carbon budget of 390 GTCO2e for the food sector, which we allocate through 2050, since most climate models reach net zero by that time.

In our benchmark calculations, this goal can be achieved in the following cases with alternatives to ASF: rapid adoption starting before 2023 with 60% of ASF replaced by 2050, rapid adoption starting 2026 with 100% of ASF replaced, and all cases between. Rapid adoption starting after 2026 or eventual replacement below 60% will not keep the food system within the 390 GTCO2e budget under our benchmark scenario (transition to 3220 Kcal per person). Transition to a plant-only diet, without alternatives to ASF, has slower adoption rates. Therefore, it must begin no later than 2025 with convergence to 100% plant-only diet by 2050 to keep the food system within 390 GTCO2e budget. The EAT-Lancet Healthy Diets scenario transition alone^24^, regardless of start date, does not remain within the 390 GTCO2e budget, even with 100% convergence and fast transition pace. This is because GHG emissions from ASF in EAT-Lancet Healthy Diets substantially exceed those of plant production^25^.

Our estimates for the reduction in GHGs resulting from the substitution of ASF are on the conservative side because we do not factor in the potential for carbon sequestration resulting from restored biomass due to reduced agricultural land use^14,26^. We opted for not including the benefits of land use change because they may take decades to manifest, whether through soil carbon sinks or reforestation, and because these benefits are highly uncertain given the broader unpredictability of the climate trajectory and other factors^26,27^.

Our calculations correspond to a reduction of global food system GHG emissions relative to the business-as-usual scenario by 33% if alternatives to ASF are used to match caloric intake, and by 31% if the replacement is with standard plant products. This impact compares favorably with proposed strategies for reducing GHG emissions from the global food system, such as major shifts of cattle production areas, feed compositions changes, and land restoration (estimated reduction of 34–85% annually)^28^. Other approaches include reducing food waste (leading to a 15% reduction in emissions from the food system) as well as yield improvements and other technological changes (1-2%)^3,29–34^.

## Results

**Fig. 1:**
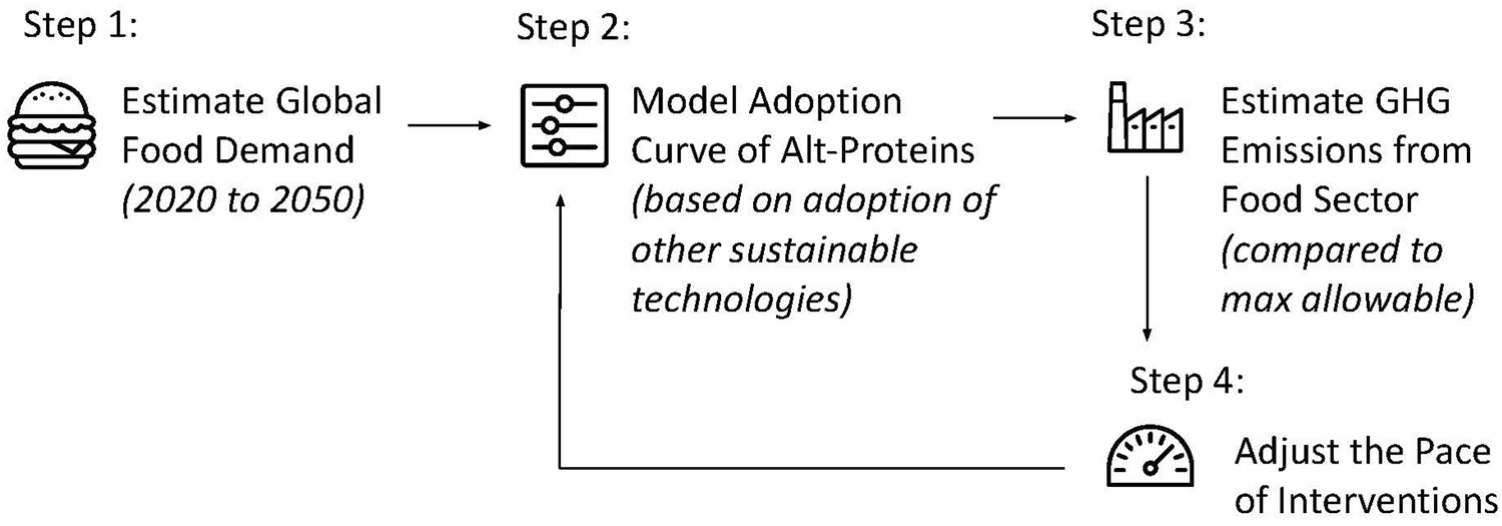
Analysis steps.

We organize our results into three main components (Fig. 1). Two factors contribute to global caloric demand: population growth and income growth. These factors together contribute to the growing demand for calories, while growing income skews the composition of food groups towards disproportionate increase in calories from ASF. This results in a substantial increase in emissions from the food system, under business-as-usual (BAU) projections, for a cumulative total of 607 GtCO2e in the 2020-2050 time period. This amount is not compatible with most estimates of the emission budget for achieving climate goals^3^.

Any changes involving consumer behavior do not occur linearly. Across a variety of products, initial adoption is slow, but it accelerates into mass adoption after a certain threshold is reached. This path is well approximated by Gompertz curves, used for modeling consumer product and technology adoption by academic researchers and marketing professionals^35–38^. For dietary changes to occur on a large scale, alternatives need to achieve parity with ASF mostly in terms of price and taste, but also satisfying additional consumer attributes. The timing of reaching this parity is key to determining cumulative food industry emissions.

Given substantially lower emissions from alternatives to ASF and from plants in general, cumulative food industry emissions can be substantially reduced by replacing ASF with alternatives or with a plant-only diet. Incremental reduction in the share of ASF in the diet, as proposed by EAT-Lancet^24^, is not sufficient to reach the needed reduction in the food sector emissions. Obviously, a combination of proposed strategies —including transition to healty diets, food waste reduction, technological improvements in agriculture, and ASF alternatives — will together yield the greatest impact^39^. However, none of the individual interventions are estimated to reduce GHG emissions as much as a rapid switch to alternatives to ASF or plant-only diets.

### Business as usual means increasing global emissions from the food industry

**Fig. 2:**
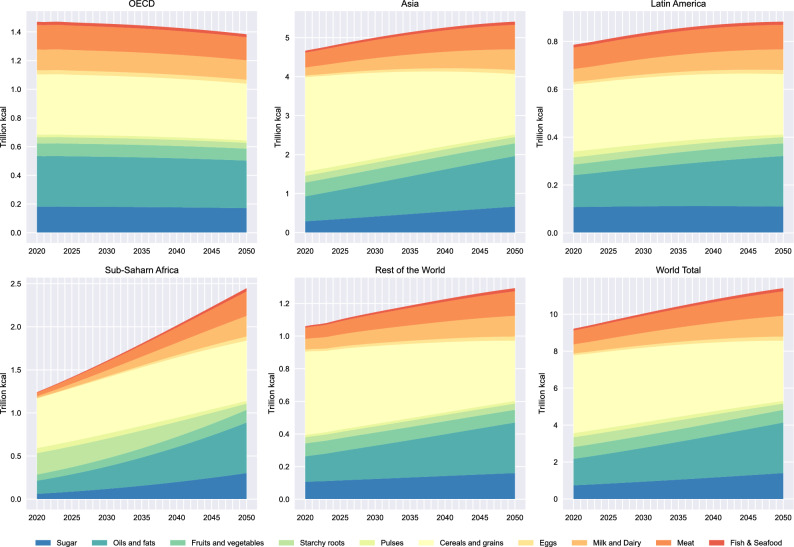
Global demand projections by food group and region: BAU. Notes: Projection of caloric demand by food group and the region. OECD includes all EU economies. Population projections are from the United Nations. Dietary projections are authors’ calculations and are based on observed trends and the estimated relationship between income and dietary compositions.

Population and income growth both contribute to the growing demand for calories. However, as incomes increase, there are also changes in diet compositions towards the current ASF-heavy “Western” diet (Fig. 2)^23^. Consumption of calorie-dense foods, including oils and fats, dairy, and meat, increases with growing income while the share of cereals and grains in the diet declines. The largest increases in both total calories and in the share of calorie-dense foods are in Sub-Saharan Africa (SSA). As a result, for the world as a whole, we estimate an increase in total calorie demand of 24% in 2050 compared to 2020, with the amount of calories obtained from all ASF (eggs, milk and dairy, meat, fish and seafood) nearly doubling — an increase of 98%, and calories from meat specifically increasing by 77%. This corresponds to an increasing share of ASF in total calories consumed from 15.6% in 2020 to 24.9% in 2050, with the share from meat products rising from 8.2% of total calories consumed in 2020 to 11.6% in 2050.

**Fig. 3:**
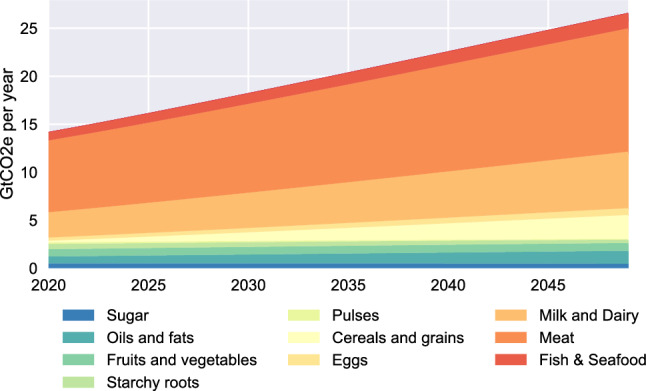
Annual emissions by food group: BAU. Notes: Annual emissions are computed based on the world diet projections in Fig. 2, with emissions by food group aggregated based on data in^25^.

Given substantially larger emissions from ASF than from plant sources — even the lowest-impact ASF GHG emissions exceed the GHG emissions of vegetable production^25^ — this change in diets results in a substantial upward trend in annual emissions from the global food system (Figure 3), doubling between 2020 and 2050, driven almost entirely by the ASF. This increase is mostly due to population growth and dietary changes, with only small portion attributable to changes in per-person calorie intake in our scenarios (see Supplement). These calculations are in line with prior results obtained using different methodologies^40^.

### Consumer choice does not follow a linear path over time

**Fig. 4:**
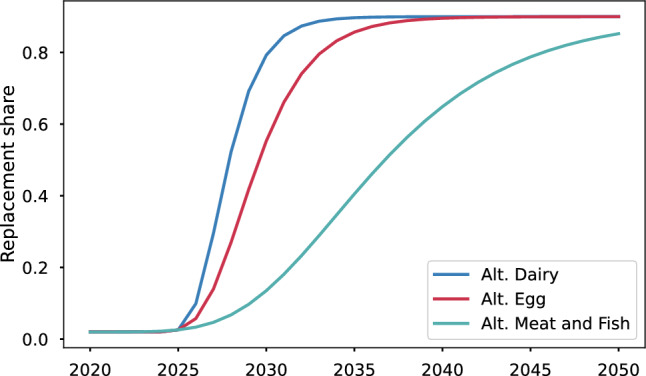
Sample dynamics of adoption of alternatives. Notes: The following equation is plotted $S_{alt j,t} = S_{alt j,2020} + FA \, \text{ e}^{\, -t^*_{j} \,\text{ e}^{-\alpha _j \, (t_j -t^*_{j})}}$, where *S* is the share of alternative *j* in year *t*, *FA* is the final replacement share (saturation level), set in this example to 0.9, growth rate constants are $\alpha _{alt. meat} = 0.18$, $\alpha _{alt. dairy} = 0.73$, $\alpha _{alt. egg} = 0.46$, which are based on the lowest, highest, and average adoption rates for solar and wind technology. Adoption start year (displacement) is $t^*_{j} = 2025$ for all food groups. Current share $S_{alt j,t}$ is 0.02 for all food groups.

There is high uncertainty about the path of consumer behavior. However, there is broad agreement in the literature that it follows a non-linear trajectory, well approximated by Gomperz curves^35–38^. Gompertz curves show that upon reaching the point at which the new product matches the key attributes of the incumbent product, there is an initial slow period of adoption, with “early adopters” helping increase awareness and scale up production, thereby lowering costs due to economies of scale. This slow initial period is followed by rapid adoption until a substantial share of replacement is reached. Once the adoption share approaches a final replacement share (or saturation level, which may be below 100%), the adoption rate slows down again. This pattern is illustrated in Fig. 4. The Figure plots, as an illustration, a case of 70% final replacement of ASF with alternatives, with the adoption start year set to 2026.

For our analysis, we vary the final replacement share and the adoption start year, but we assume throughout that the adoption pace is fastest for alternative milk and dairy, medium pace for alternative eggs, and slow for meat and fish, as reflected in Fig. 4. We conduct a sensitivity analysis for the adoption pace parameter for each food group by varying the growth rate parameter by a factor of two in either direction, shown in the Supplement.

### Transition timing is key to cumulative emissions

**Fig. 5:**
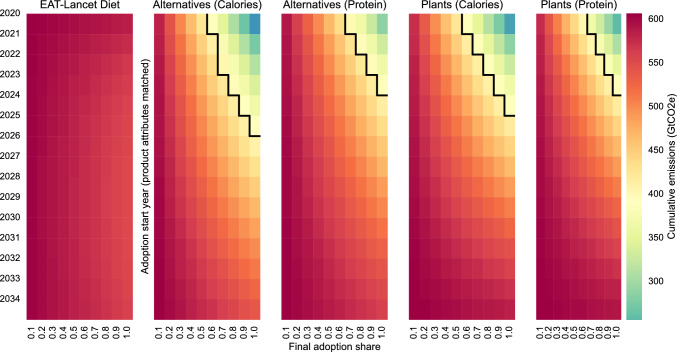
Cumulative emissions in different scenarios as a function of final adoption share and adoption start year. Cumulative emissions are computed for 2020-2050 time period, but only 2020-2035 time period is plotted given the focus on adoption start year. EAT-Lancet is the convergence to EAT-Lancet diets globally. Alternatives (Calories) is the replacement of ASF with alternatives to match caloric value; Alternatives (Protein) is the replacement of ASF with alternatives to match the total protein demand of the BAU scenario. Plants (Calories) is the replacement of ASF with plants to match caloric value; Plants (Protein) is the replacement of ASF with plants to match protein demand in the BAU scenario. The adoption start year corresponds to the year in which the attributes of incumbent products are matched by alternatives. The scenario plotted is based on no adoption delay and slow introduction of cultured and fermentation-based alternatives to ASF to allow for various adoption barriers^41^. The final adoption share is the 2050 target. Healthy Diets transition is assumed to have a fast transition parameter $\alpha _{HD} = 0.73$, while the transition to plant-only diet is assumed to be slow with $\alpha _{Plants} = 0.18$^42–44^. The center color of the color bar is set to 390 GtCO2e, the estimated emission budget for the food industry, and is the same across panels. The black line approximately separates the area below and above this budget.

Cumulative emissions from the food industry crucially depend on the path of the transition (Fig. 5). Even a fast transition to 100% EAT-Lancet Healthy Diets starting in 2024 results in cumulative emissions exceeding 500 GtCO2e (a 22% decline relative to BAU)^24^. By most estimates, this is still not compatible with reaching climate goals, which allocate 390 GtCO2e to the food sector; therefore, more substantial substitution away from ASF is required.

To stay within the 390 GtCO2e budget, the scenario with only plant-based alternatives to ASF through 2032 and cultured and fermentation-based alternatives increasing to 27% of all alternatives by 2050 (mostly PB), requires 60% replacement of ASF with alternatives by 2050 if attribute parity was reached in 2023, and 100% replacement if adoption start is delayed until 2026 (2025, if the protein demand rather than caloric demand is matched). Over time, technological innovation may increase protein content in staple crops used for alternatives to ASF, but we do not incorporate this into our calculations because of high degree of uncertainty. Any delay beyond 2026 means that a switch to alternatives alone will not be sufficient to satisfy global food demand without exceeding the cumulative emission budget. However, the climate goals can still be accomplished if adoption of alternatives is accompanied by the reduction in food waste, which we do not factor in, or if diets converge to those proposed by EAT-Lancet in addition to the adoption of alternatives, as shown in the Supplement.

Because of the slower transition to plant-only diets without alternatives to ASF, 80% of ASF need to be replaced with plants by 2050 if the transition starts before 2024 to stay within the 390 GtCO2e budget (90% to match protein rather than caloric demand). Delaying the start of transition to plant-only diets until after 2025 will result in exceeding the emission budget even with 100% plant-only diets globally by 2050.

## Discussion

Our results show that alternatives to ASF, if they are developed or improved to satisfy consumer preferences, can substantially reduce GHG emissions from the food system to levels consistent with global climate goals.

### Adoption of alternatives to ASF leads to larger GHG emission reduction than other approaches

**Fig. 6:**
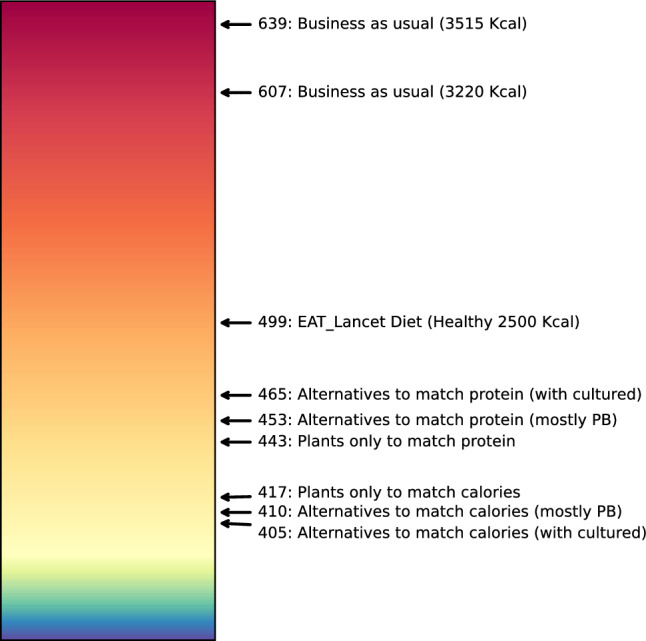
Cumulative emissions from the food system: 2020-2050. Notes: This figure shows cumulative emissions from the food system between 2020 and 2025. Annual emissions are computed based on the world diet projections in Fig. 2, and transition to alternatives to ASF or plant-only diet taking off in 2025 and targeting 90% by 2050. Corresponding transition curves are shown in Fig. 4.

Replacing all animal-source foods with alternatives by 2050 without changing the composition of the average diet can reduce food system emissions from 607 Gt CO2e to 405 Gt CO2e in our benchmark scenario (a 202 Gt CO2e reduction, Figure 6). This is a substantially larger reduction than can be achieved by food waste reduction, yield improvements, a shift toward plant-rich diets (65-92 Gt CO2e in Project Drawdown calculations), or the 108 Gt CO2e impact from a transition to EAT-Lancet Healthy Diets in our calculations without a shift to alternatives^24,34^. Thus, alternatives to animal-source foods are likely the most promising path to food system sustainability. Importantly, these reduction calculations are based on 90% replacement of animal-source foods by 2050 with transition starting in 2025. Because cumulative emissions are key, delays in matching the attributes of incumbent ASF products and demand take-off, or limited ultimate replacement share, reduce the impact substantially. Thus, innovation in alternatives to ASF needs to be rapid and supported by a strong regulatory environment and investment from both the private and public sectors. In particular, policies similar to those that supported the development of alternative energy sources are necessary for a rapid transition to a sustainable food system.

It is clear that shifting global diets away from ASF is key to reaching climate goals. Moreover, the change needs to be sufficiently drastic: incremental changes — such as shifting to a healthier diet in developed countries — are not likely to be sufficient due to the continuing growth of ASF consumption in developing countries driven by continuing population growth and increasing wealth^23^. Can this shift occur without new technologies and products offering alternatives to ASF? It is possible, but the pace of transition to predominantly plant-based diets is likely to be insufficient: our calculations show that the transition must begin immediately and reach 100% plant-based consumption globally by 2050. Given current trends toward a rising ASF share, this is not likely^42–44^.

### Policy interventions needed to speed up the adoption of alternatives to ASF

Alternatives to ASF are designed to make the transition away from ASF more feasible for consumers by minimizing behavior change and instead substituting new technologies for ASF inputs — plant-based or cultured (including fermentation-based). This approach enables faster adoption and makes climate-consistent scenarios more realistic. Even with alternatives to ASF, however, the shift is unlikely to begin soon enough without intervention. Our adoption curve parameters are based on the transition to alternative energy, which received substantial global government support. Now that the pace of adoption of electric vehicles and solar panel generation is exceeding forecasts^45^, policy interventions to help jump-start the transition away from ASF are likely to be highly impactful for reaching climate goals. Such interventions could include both supply-side incentives, such as research grants and R&D subsidies for quality improvement and subsidized loans to enable rapid scaling that meets growing demand, and demand-side measures, such as incentives for food companies, retailers, and the food service industry to reduce their climate impact.

### Other considerations beyond the scope of this study

It is worth reminding the reader that we do not include in our calculations CO2 sequestration through the restoration of ecosystems on the land freed from pasture use and animal feed production, which could be as high as 332-547 GtCO2 by 2050, potentially making cumulative GHG emissions from the land currently used for agriculture negative on net^14,46^. If these effects are added, there is potential for reaching climate goals with lower final replacement of ASF^15,47^. That said, the exact timeline of the carbon sequestration potential prior to 2050 is highly uncertain, which is why we are not including these considerations in our benchmark analysis.

We are focusing on reaching global nutrition goals in terms of caloric and protein intake. Thus, an implicit assumption we make is that micronutrient goals can be achieved with a reduced share of ASF. This assumption is supported by a number of recent studies that show that diets that rely on alternatives to ASF or just plants are able to match nutritional needs along most dimensions^48^. While not all necessary nutrients can be obtained from plants, most notably, vitamin B-12, certain omega-3s, as well as taurine, cultured and fermented alternatives are able to provide these, and technologies are available to synthesize these as supplements^49^.

We rely on the most commonly cited estimates of emissions by food group^25^, which means that we do not explicitly take into account recent discussions around limitations of relying on CO2e aggregate without explicitly accounting for the timing of the emissions’ impact on climate^50^. This concern is equally important for emissions arising from both the food system and the energy sector, as these are the two main sources of CH4 emissions^51^, which have a different global warming potential than CO2.

In this analysis, we do not address the financial aspects of diet transitions. Alternatives to ASF are still in early stages of development and require substantial investment. In the long run, given the lower demand for resources by plants and alternatives to ASF compared to animal agriculture, a climate-consistent dietary transition is likely to reduce overall food production costs. This means that the initial investment may pay for itself over time. The analysis of the financial costs of this abatement strategy and its comparison with other emission-reduction approaches could be conducted along the lines of marginal abatement cost curves^52^, but is beyond the scope of the current study.

Finally, we do not analyze the three other important environmental costs that are due to animal agriculture: land use and degradation, water depletion and pollution, and biodiversity loss^46,53,54^. These costs can lead to even more immediate negative effects than GHG emissions, thus underscoring the need for rapid shift away from ASF. They arise due to the ultimate inefficiency of the animal production technology for energy conversion^55^. This inefficiency also has a social cost: high share of ASF in high-income countries’ diet increases global demand for feed crops such as soy, corn, and wheat, likely raising global price of these staple crops and thus making them less accessible to low-income consumers.

## Methods

As discussed in Section 1, we have structured the analysis into three components. First, we estimate the global calorie consumption by food group between 2020 and 2050, then we model the adoption of alternatives to animal-source food categories, including both pure plant and manufactured alternatives. Finally, we calculate the GHG emissions under different scenarios.

### Global demand scenarios to project total food consumption paths

We estimate global food demand using population forecasts, daily per capita caloric intake, and estimates of the share of caloric intake sourced from proteins.

We segregate food consumption into food groups based on their sources — plants, eggs, dairy, fish, animal-source meat, and alternative meat. Plants consist of the following sub-categories: sugar, oils and fats, fruits and vegetables, starchy roots, pulses, cereals, and grains. We quantify the weight of each source through analysis of existing dietary patterns using Food and Agriculture Organization of the United Nations (FAO) individual consumption data and National Geographic “What the World Eats” data.

We first calculate 2020 caloric consumption by food group by country using FAO individual consumption data by country, then aggregate this data into five regions with distinct consumption patterns: OECD90+EU, Asia, Latin America, and Sub-Saharan Africa. Other countries are grouped into “Other” category.

For the projections, we consider a 30-years time frame, from 2020 until 2050. This is the same time frame considered by the IPCC’s assessment of the emissions budget required to limit temperature rise to $1.5^{\circ }$C^56^. We model global food demand (GFD) for each year *t*, focusing on main food sources indexed *j*.

1 $$\begin{aligned} GFD_{jt} = 365 \, N_t \, C_t \, S_j. \end{aligned}$$

For $C_t$, daily caloric intake per person, we consider two scenarios. Our benchmark scenario assumes that there is a global convergence in both the average daily calories consumed per individual and the food group breakdown to the 2020 OECD90+EU numbers. In other words, for OECD90+EU the overall calories per individual and food group breakdown remain unchanged between 2020 and 2050, while for other regions the calories per individual and breakdown by food source converge to the same numbers as for OECD90+EU. Overall, this means that the global average caloric intake per person per day increases from 3220 kcal in 2020 to 3515 kcal in 2050. An argument can be made, however, that convergence to 3515 kcal per person per day globally is inconsistent with healthy living and might exaggerate global demand. Instead, as a benchmark, we consider convergence to the current global average of 3220 kcal per person per day. We conduct a sensitivity analysis with respect to this assumption by using the 3515 kcal target, reported in the Supplement.

We also consider the EAT Lancet “Healthy Diet” scenario which prescribes per person caloric intake of 2500 kcal and a different composition of food groups, resembling what is commonly referred to as “mediterranean” diet^24,57,58^. We construct trends for shares of ASF to converge by 2050 to upper limits for animal-source products (eggs, dairy, meat) recommended by EAT Lancet as well as for other shares of food groups. We consider transition to this diet, following realistic consumer adoption dynamics, as an alternative to replacement of ASF. In addition, we consider a conter-factual linear transition to EAT Lancet diet combined with adoption of alternatives to ASF, reported in the Supplement.

Global caloric demand is the product of global population projections and per-person caloric intake for each food group. The global population is from the United Nation’s projections for medium fertility and constant mortality scenarios (published by the Department of Economic and Social Affairs) and grows to 9.71 billion in 2050 and is evaluated as of July 1 for each year.

In 2020, around 82% of the global caloric demand was met by plants. Animal-source meat provided 8%, dairy provided 7%, and fish provided 1% of the global caloric requirements. Overall consumption of alternative animal-source foods is negligible in 2020 and is modeled as 2%.

### Two scenarios for the shift to alternatives to animal-source foods

To project the shift in consumption from animal-source foods to alternative sources, we develop a model of consumer adoption for plant-based eggs, plant-based milk and dairy, plant-based meat and fish, and cultured meat and fish.

We model the shift from animal-source foods to alternative protein sources from the initial market share of alternatives observed in 2020 to shares varying from 0 to 100% by 2050. To model transition dynamics, we rely on the Gompertz adoption model, which is commonly used for modeling new product adoption dynamics^35–38^.

Gompertz adoption model allows us to create dynamic scenarios for alternative sources replacing animal-source foods:

2 $$\begin{aligned} S_{alt j,t} = S_{alt j,2020} + FA \, \text{ e}^{\, -t^*_{j} \,\text{ e}^{-\alpha _j \, (t_j -t^*_{j})}} , \end{aligned}$$

where $S_{alt j,t}$ is the share of food type *j* substituted for an alternative in year *t*, $S_{alt j,2020}$ is such share observed in 2020, *FA* is final adoption share (saturation level), $\alpha _j$ is the growth rate constant that drives the speed of adoption, $t^*_j$ is the year when alternative source achieves perceived taste, texture, other attributes, and price parity (we refer to it as “adoption start year”).

Since alternative foods are still an emerging product and technology, we rely on previous technology adoption experience to calibrate the speed of adoption $\alpha _j$ of an alternative for each product category. In particular, we rely on observed adoption speed for recently matured sustainable technologies in energy generation, wind and solar^35^. Parameters from the energy transition are applicable because, similar to alternative food sources, these are products and technologies that are not entering a “white space” in consumer demand. These technologies provide an alternative way of satisfying consumer needs now satisfied with other products, with the purpose of achieving a sustainability goal, that is an externality for consumers as well as producers. As with energy transition, early adopters are willing to pay extra due to non-pecuniary considerations, but mass adoption requires a parity of attributes with incumbent products to be reached first, after which adoption accelerates rapidly.

We choose the following values for our benchmark scenario: $\alpha _{alt. meat} = 0.18$, $\alpha _{alt. dairy} = 0.73$, $\alpha _{alt. egg} = 0.46$, which are the lowest, highest, and average adoption rates for solar and wind technology. We test sensitivity of the model results to these parameters in the Supplement. We set the current market share for alternative products at 2%.

Currently, there are two main types of technologies for producing analogs to animal-source foods: plant-based alternatives, which are broadly available in some countries for a number of animal-source product categories, and “high-tech” alternatives that include cultured alternatives (also known as cultivated alternatives or cellular agriculture), which focus primarily on meat and fish products, precision fermentation and molecular farming, targeting meat as well as dairy products, and biomass fermentation, again focusing on meat and fish alternatives^48^. Some precision fermentation products are available with very limited distribution, while cultured alternatives are not generally available.

For our scenario building, we combine all high-tech alternatives into one group — “cultured” alternatives, because of their similar impact on GHG emissions^19,20^. We keep plant-based technologies separate. For our analysis, we construct two scenarios for the composition of alternatives to animal food sources: rapid shift to cultured (RS) scenario, which is based on projections in which consumer adoption of cultured products starts in 2026 and reaches two-thirds of the alternative meat market by 2050;^59^ mostly plant-based (PB) scenario, instead, keeps the share of plant-based alternatives at 100% until 2033 and then assumes a quarter of an annual rate of increase of cultured alternatives in RS scenario.

### GHG emissions by food group

We estimate the total reduction in annual GHG emissions from food consumption due to the introduction of alternative protein sources, based on adoption scenarios and emissions factors per food source.

To translate food demand into emissions impact, we compute emission factors in terms of CO2e GHGs per 1000 Kcal or per 100g of protein for each type of food (e.g. grains within plants)^25^. Using these factors for individual food items within each food source category, we aggregate them to the food source level as a weighted average to derive total category emissions factors reported in Table 1. We then aggregate emissions by food source to compute total emissions from food consumption.

For the GHG emissions from plant-based alternatives to egg, dairy, and meat, we compute a weighted average of plants that are ingredients in such alternatives. Implicitly, this assumes clean energy sources for food processing up to the stage that is equivalent to animal-source foods^22^. For cultured alternatives, we use mid-range values of recent estimates of GHG emissions per 1000 Kcal or 100g of protein^60,61^, which is substantially higher than the estimates in earlier studies^62^. This value of GHG emissions is higher than projected in recent LCA reports by cultivated meat companies. Due to high uncertainty of these estimates, we conduct sensitivity analysis by doubling and tripling this value; see Supplement.

**Table 1:** GHG Emissions by Food Source (kg CO2e)

	Sugar	Oils & fats	Fruits & vegetables	Starchy roots	Pulses	Cereals & grains	ASF				Plant-based			Cultured Meat/Fish
							Eggs	Dairy	Meat	Fish	Eggs	Dairy	Meat/Fish	
per 100g protein	$\cdot$	$\cdot$	5.4	7.2	0.6	3.5	4.2	9.7	13.6	7.2	1.6	1.8	1.6	2.5
per 1000 kcal	0.7	0.5	1.2	1.0	0.4	0.8	3.2	5.4	9.9	9.5	0.7	0.7	0.8	1.2

Notes: Kg CO2e GHG emissions per 1000 Kcal or per 100g of protein for each food source^25,60^.

We use two scenarios for the substitution of animal-source foods with alternatives. The first is a caloric equivalent (CE) scenario with GHG emissions based on substituting alternatives for animal-source foods 1-to-1 in terms of total calories. The second is the protein equivalent (PE) scenario based on substituting alternatives for animal-source foods 1-to-1 in terms of total protein. While protein can be obtained from a variety of plants, a common perception is that animal-source foods are an important source of protein. Indeed, the ratio of protein to calories in animal-source foods is generally higher than that of plants, even though some novel plant-based alternatives to ASF have higher protein per calorie ratio than ASF they are replicating. To reflect this, we convert GHG emissions per Kcal into GHG emissions per gram of protein and keep the total protein consumption in the alternative sources scenarios the same as in business-as-usual scenarios. This results in a higher amount of alternatives in total consumption compared to the caloric equivalent model.

### Carbon budget assumptions

There are a wide variety of estimates of the total carbon budget that remains to achieve $1.5^{\circ }$C with a probability of 67% or 50%^63^. Given limited progress on emission mitigation, we instead look at the 2-degree goal with 67% probability.

Our ultimate question is how much GHG emission savings from the food system can we achieve by substituting ASF with alternatives. It is best to answer this question in the context of the total carbon budget available to the food system. This number relies on two assumptions with a high degree of uncertainty: the exact magnitude of the impact of GHG concentration on temperature change is fundamentally uncertain; transition paths of other emission sources, especially energy and transportation, are subject to multiple sources of uncertainty. With this in mind, we invite the reader to use their own preferred estimate for the carbon budget that we can allocate to the food system in the coming decades. Our preferred estimate is 390 GtCO2e which corresponds to a third of the total GHG budget of 1170 GtCO2e which we take from IPCC6. This is the budget to contain global warming to 2 degrees C with 67% probability. We extrapolate the estimate that, in 2015, the food system contributed 34% to GHG emissions, to arrive at the food sector budget of 390 GtCO2e^2^. While the CO2 budget is usually reported between 2020 and 2100, most of the paths underlying the calculations have net zero achieved by 2050 followed by net negative emissions. Thus, we allocate the entire budget between 2020 and 2050.

## Supplementary Information

Supplementary Information.

## Data Availability

Calculations for the projections in this analysis are conducted in Python and Excel are shared on Github along with necessary data inputs. https://github.com/GalinaHale/FoodClimate.
