# Supplementary material for: Pace of adoption of alternatives to animal-source foods is an important factor in reaching climate goals
Source: Sci Rep. 2025 Jul 2;15:22643. doi: 10.1038/s41598-025-07866-x (PMC12217607; doi:10.1038/s41598-025-07866-x)
Supplement: Supplementary file 1 — Supplementary Information. [file 41598_2025_7866_MOESM1_ESM.pdf]

Pace of adoption of alternatives to animal-source foods is an important factor in reaching climate goals

Galina Hale[a*] Vlad Oncescu[b] Ritesh Bhangale[b]

[a]UCSC, NBER, CEPR

[b]Accenture

May 27, 2025

Supplement: Sensitivity to assumptions

We conduct sensitivity analysis to show how our assumptions affect our results.

1 Alternative caloric target for 2050

In our benchmark we set the target for total per person per day caloric intake for all regions 3220, which is the current average caloric consumption in the world. This implies actual reduction in caloric intake in OECD+EU countries. This caloric consumption, however, is not consistent with recent trends. If the recent trend continues, the daily per person consumption will grow in OECD+EU countries to 3515Kcal by 2050. As an alternative, we set a target of 3515 for all countries, meaning that per person consumption grows in all countries. Not surprisingly, this change increases GHG emissions in all scenarios, but does not change the results qualitatively, as shown in Figure 1. As before, switching to EAT-Lancet Healthy Calories scenario does not produce sufficient emission reduction. In other scenarios, minimum ultimate adoption share for a given adoption start year is about 10 percentage points higher than in the benchmark.

What if we combine a linear transition to EAT-Lancet Healthy Calories scenario with the switch to alternatives to ASF or plants. While we do not believe this scenario is likely, because any diet change is gradual and non-linear, we show it here as a counterfactual calculation. Since the EAT-Lancet diet includes a lower share of protein than BAU and overall lower calorie intake, the GHG emissions from the food sector can stay within the budget with a lower share of ASF replaced and

*Corresponding author: gbhale@ucsc.edu. We thank three anonymous referees, Chris Barrett, Erin Rees Clayton, and Irene Monasterolo for the valuable feedback. All errors are ours. The views in this paper are solely of the authors and do not necessarily reflect the views of Accenture, NBER, or CEPR.

Figure 1: Cumulative emissions in different scenarios as a function of final adoption share and adoption start year: 3515 calories target

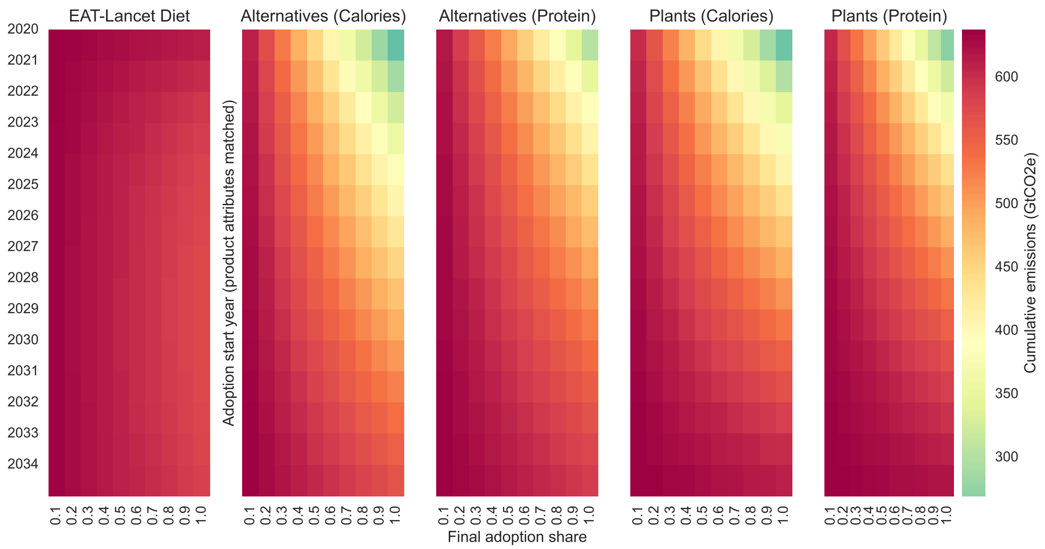

Cumulative emissions are computed for 2020-2050 time period. Instead of targeting 3220Kcal per person per day, these calculations target 3515Kcal per person per day globally by 2050. Alternatives (Calories) is the replacement of ASF with alternatives to match caloric value; Alternatives (Protein) is the replacement of ASF with alternatives to match the total protein demand of the BAU scenario. Plants (Calories) is the replacement of ASF with plants to match caloric value; Plants (Protein) is the replacement of APS with plants to match protein demand in the BAU scenario. The adoption start year corresponds to the year in which the attributes of incumbent products are matched by alternatives. The scenario plotted is based on no adoption delay and slow introduction of cultured and fermentation-based alternatives to ASF to allow for various adoption barriers.(1) The final adoption share is the 2050 target. Healthy Diets transition is assumed to have a fast transition parameter $\alpha_{HD} = 0.73$, while the transition to plant-only diet is assumed to be slow with $\alpha_{Plants} = 0.18$.(2; 3; 4) The center color of the color bar is set to 390 GtCO2e, the estimated emission budget for the food industry, and is the same across panels.

more delayed adoption (Figure 2). If the calorie content of the diet is to be matched, 80% of ASF need to be replaced by 2028.

2 Decomposition of consumption growth and an alternative BAU scenario

In our analysis, business-as-usual (BAU) calculations include a combination of three factors: population growth, changes in caloric intake per person per day, which vary by region, and changes in average composition of these calories across food groups, as rising wealth in highly-populated regions leads to their diets converging to those in the OECD+EU benchmarks. Figure 3 shows the decomposition of benchmark emissions per year per food group (first panel) into these three components (middle three panels). The final panel goes back to the benchmark but sets the final caloric target to 3515 calories per person per day instead of benchmark 3220, as described in the

Figure 2: Cumulative emissions in different scenarios as a function of final adoption share and adoption start year combined with linear transition to EAT-Lancet diet

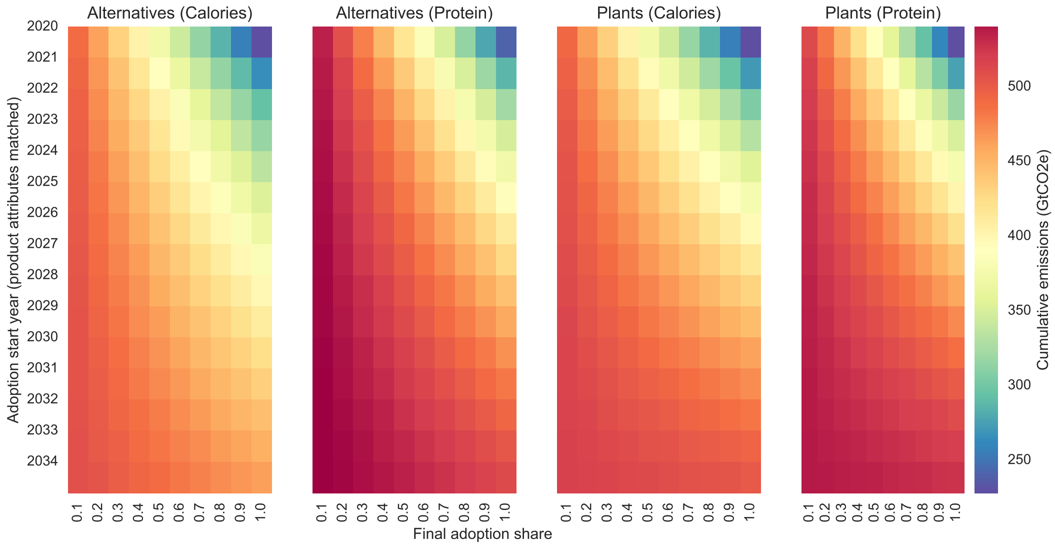

Cumulative emissions are computed for 2020-2050 time period. Instead of targeting 3220Kcal per person per day with the current macronutrient composition, EAT-Lancet diet composition and total calorie intake is set as a target for global demand by 2050. Alternatives (Calories) is the replacement of ASF with alternatives to match caloric value; Alternatives (Protein) is the replacement of ASF with alternatives to match the total protein demand of the BAU scenario. Plants (Calories) is the replacement of ASF with plants to match caloric value; Plants (Protein) is the replacement of APS with plants to match protein demand in the BAU scenario. The adoption start year corresponds to the year in which the attributes of incumbent products are matched by alternatives. The scenario plotted is based on no adoption delay and slow introduction of cultured and fermentation-based alternatives to ASF to allow for various adoption barriers.(1) The final adoption share is the 2050 target. Healthy Diets transition is assumed to have a fast transition parameter $\alpha_{HD} = 0.73$, while the transition to plant-only diet is assumed to be slow with $\alpha_{Plants} = 0.18$.(2; 3; 4) The center color of the color bar is set to 390 GtCO2e, the estimated emission budget for the food industry, and is the same across panels.

previous section. We can see that both population growth and diet change contribute substantially to the increase in emissions. The increase in caloric intake is less important in magnitude.

We can recompute cumulative emissions for 2020-2050 time period in each of these cases. These figures are reported in each panel of Figure 3. As we described, in the benchmark, BAU scenario produces 607 GtCO2e. Without population growth, this number is 554, which means that population growth contributes 85 GtCO2e. Without diet change, cumulative emissions are 500 GtCO2e, which means diet change contributes 109 GtCO2e. Finally, if we combine both factors, but replace the caloric target with 3515 goal, cumulative emissions will be only slightly higher than in the benchmark, 639 GtCO2e.

Figure 3: Annual emissions by food group: decomposition and alternative scenarios

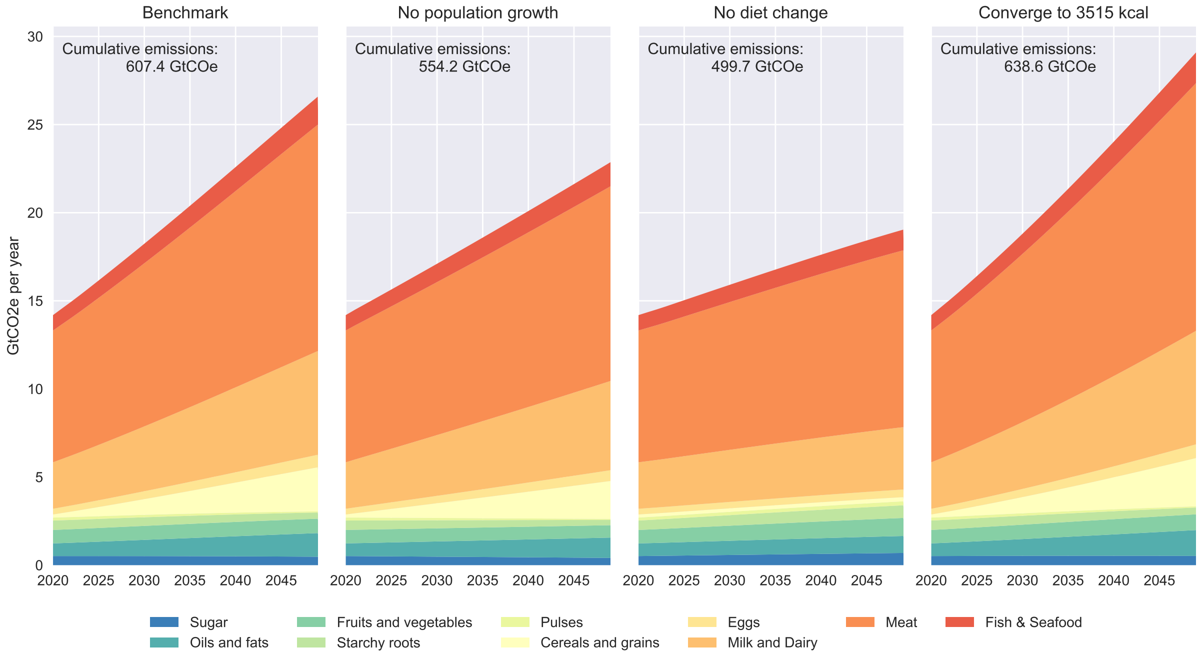

Notes: Benchmark annual emissions are computed based on the world diet projections, with emissions by food group aggregated based on data in (5). The first panel is the benchmark, for comparison. The two middle panels decompose overall dynamics by shutting down one factor at a time: population growth and change in diets. The last panel reports emissions that target 3515 calories per person per day globally by 2050 instead of 3220 in the benchmark. Cumulative emissions are reported in each panel.

3 Alternative diet adoption parameters

Our results do not change substantially when we replace the assumption of mostly plant-based alternatives to ASF to rapid adoption of cultured alternatives (Figure 4). The share of cultured alternatives does not make much impact on the calculations due to similar emissions from plant-based and cultured alternatives to ASF.

Returning to the benchmark specification, we also explore sensitivity to the Gompertz curve shape parameter α. In Figures 5-6 we report two modifications: all α's are reduced by half and all α's are doubled. We find that substantially slowing the adoption has a dramatic effect on the carbon budget implications with the only way for the food system to stay within 390GtCO2e is immediate adoption and eventual adoption share in excess of 80%. In contrast, speeding up the adoption rate only has a small improvement of the overall carbon footprint of the food system.

Figure 4: Cumulative emissions in different scenarios as a function of final adoption share and adoption start year: rapid shift to cultured alternatives

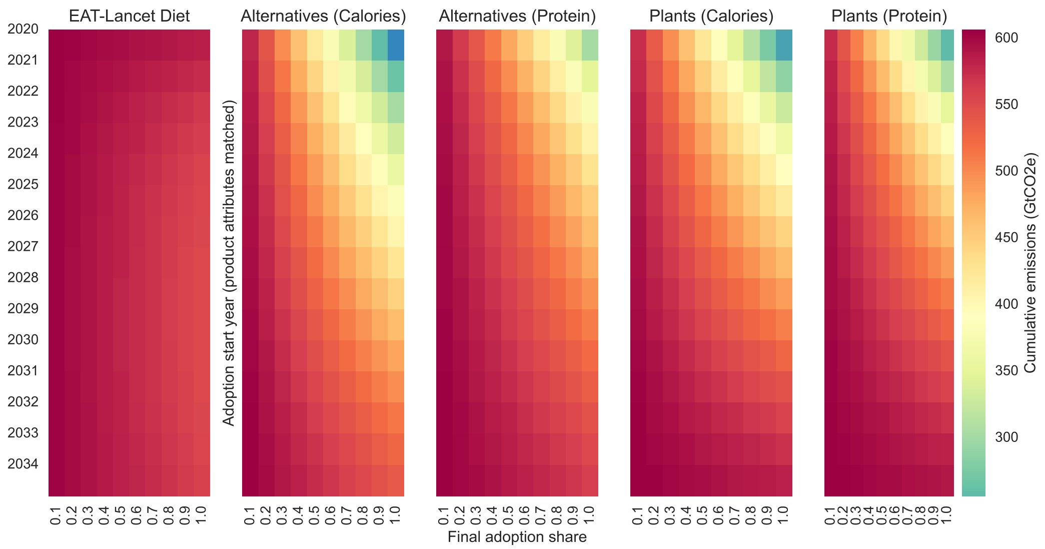

Cumulative emissions are computed for 2020-2050 time period. EAT-Lancet is the convergence to EAT-Lancet diets globally. Alternatives (Calories) is the replacement of ASF with alternatives to match caloric value; Alternatives (Protein) is the replacement of ASF with alternatives to match the total protein demand of the BAU scenario. Plants (Calories) is the replacement of ASF with plants to match caloric value; Plants (Protein) is the replacement of APS with plants to match protein demand in the BAU scenario. The adoption start year corresponds to the year in which the attributes of incumbent products are matched by alternatives. The scenario plotted is based on no adoption delay and fast introduction of cultured alternatives to ASF to allow for various adoption barriers (1). Final adoption share is the 2050 target. Healthy Diets transition is assumed to have fast transition parameter $\alpha_{HD} = 0.73$, while transition to plant-only diet is assumed to be slow with $\alpha_{Plants} = 0.18$ (2; 3; 4). The center color of the color bar is set to 390 GtCO2e, estimated emission budget for the food industry and is the same across panels.

4 Changing GHG emissions from cultivated alternatives

We realize that there is not yet a consensus for the estimates of emissions from cultivated alternatives to ASF, since the industry has not yet scaled up and it is unclear what energy inputs it will use going forward. In addition to our main sources, (6)(7) we reviewed sustainability reports of a number of California-based cultivated meat companies and our assumptions are on the high end compared with their projections as well as compared to prior studies.(8) Hypothetically, if emission-free energy is used in the production process, the GHG emissions from this technology could be very minimal.

The risk to our estimates comes from the possibility that emissions from cultivated alternatives are higher than we model. Thus, we explore the sensitivity of our results to the parameterization reported in the paper. In particular, we report our analysis under both slow and fast adoption of cultivated alternatives, while doubling and tripling emissions from these sectors. Figures 7 and 8 report the results. We can see that the results are not particularly sensitive to our parameterization

Figure 5: Cumulative emissions in different scenarios as a function of final adoption share and adoption start year: adoption rate twice as slow

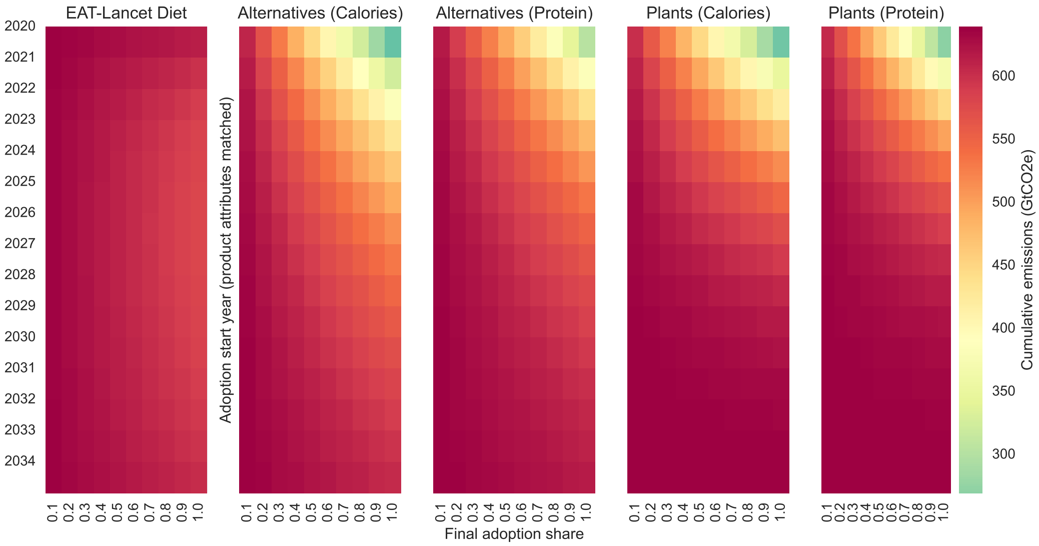

Cumulative emissions are computed for 2020-2050 time period. EAT-Lancet is the convergence to EAT-Lancet diets globally. Alternatives (Calories) is the replacement of ASF with alternatives to match caloric value; Alternatives (Protein) is the replacement of ASF with alternatives to match the total protein demand of the BAU scenario. Plants (Calories) is the replacement of ASF with plants to match caloric value; Plants (Protein) is the replacement of APS with plants to match protein demand in the BAU scenario. The adoption start year corresponds to the year in which the attributes of incumbent products are matched by alternatives. The scenario plotted is based on no adoption delay $\delta = 0$ and slow introduction of cultured alternatives to ASF to allow for various adoption barriers (1). Final adoption share is the 2050 target. All transition parameters are halved compared to the benchmark. Thus, Healthy Diets transition is assumed to have faster transition parameter $\alpha_{HD} = 0.365$, while transition to plant-only diet is assumed to be slowest with $\alpha_{Plants} = 0.09$. The center color of the color bar is set to 390 GtCO2e, estimated emission budget for the food industry and is the same across panels.

of emissions from cultivated alternatives.

Figure 6: Cumulative emissions in different scenarios as a function of final adoption share and adoption start year: adoption rate twice as fast

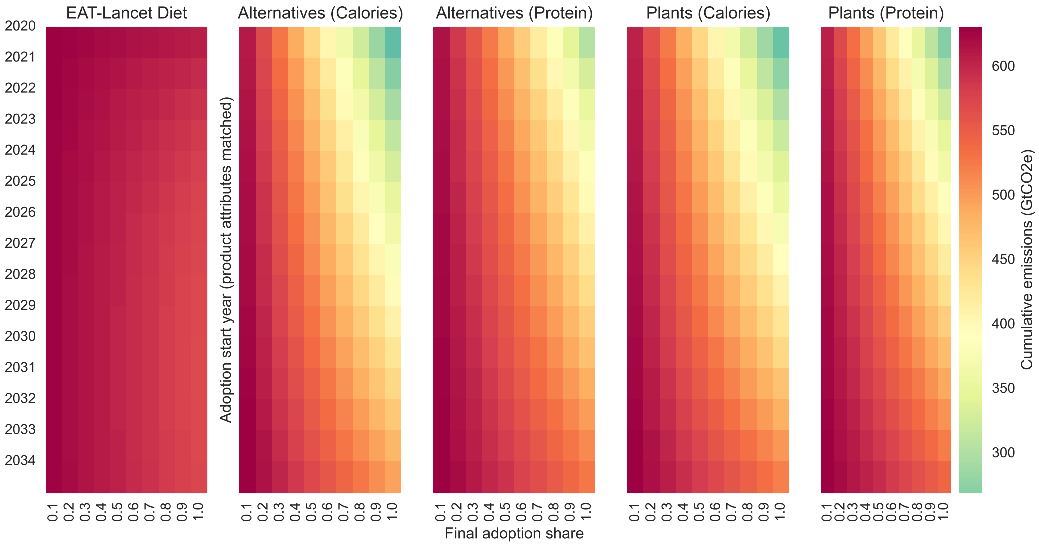

Cumulative emissions are computed for 2020-2050 time period. EAT-Lancet is the convergence to EAT-Lancet diets globally. Alternatives (Calories) is the replacement of ASF with alternatives to match caloric value; Alternatives (Protein) is the replacement of ASF with alternatives to match the total protein demand of the BAU scenario. Plants (Calories) is the replacement of ASF with plants to match caloric value; Plants (Protein) is the replacement of APS with plants to match protein demand in the BAU scenario. The adoption start year corresponds to the year in which the attributes of incumbent products are matched by alternatives. The scenario plotted is based on no adoption delay $\delta = 0$ and slow introduction of cultured alternatives to ASF to allow for various adoption barriers (1). Final adoption share is the 2050 target. All transition parameters are doubled compared to the benchmark. Thus, Healthy Diets transition is assumed to have fastest transition parameter $\alpha_{HD} = 1.46$, while transition to plant-only diet is assumed to be slower with $\alpha_{Plants} = 0.36$. The center color of the color bar is set to 390 GtCO2e, estimated emission budget for the food industry and is the same across panels.

Figure 7: Cumulative emissions in alternatives to ASF scenarios as a function of final adoption share and adoption start year: double emissions from cultivated alternatives

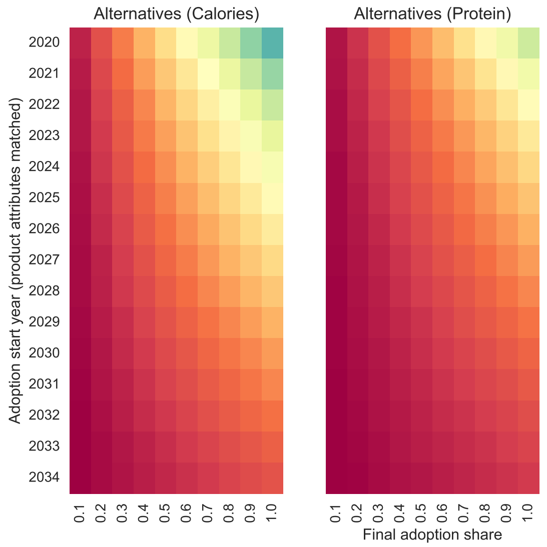

(a) Fast adoption of cultivated

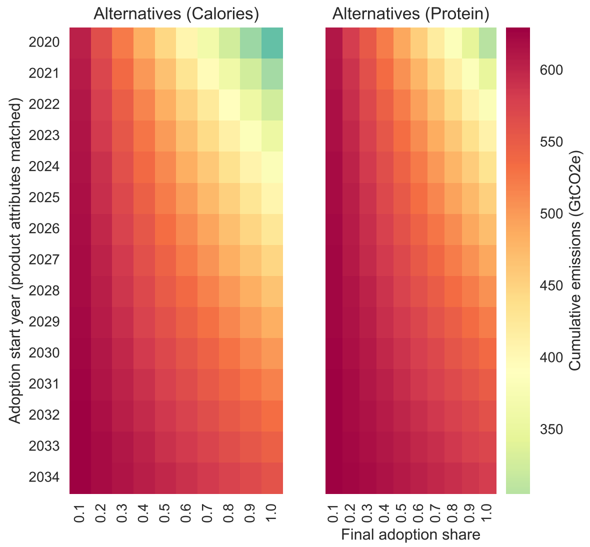

(b) Slow adoption of cultivated

Cumulative emissions are computed for 2020-2050 time period. Alternatives (Calories) is the replacement of ASF with alternatives to match caloric value; Alternatives (Protein) is the replacement of ASF with alternatives to match the total protein demand of the BAU scenario. The adoption start year corresponds to the year in which the attributes of incumbent products are matched by alternatives. The scenario plotted is based on no adoption delay $\delta = 0$ and fast or slow introduction of cultured alternatives to ASF, as indicated. Final adoption share is the 2050 target. The center color of the color bar is set to 390 GtCO2e, estimated emission budget for the food industry and is the same across panels.

Figure 8: Cumulative emissions in alternatives to ASF scenarios as a function of final adoption share and adoption start year: triple emissions from cultivated alternatives

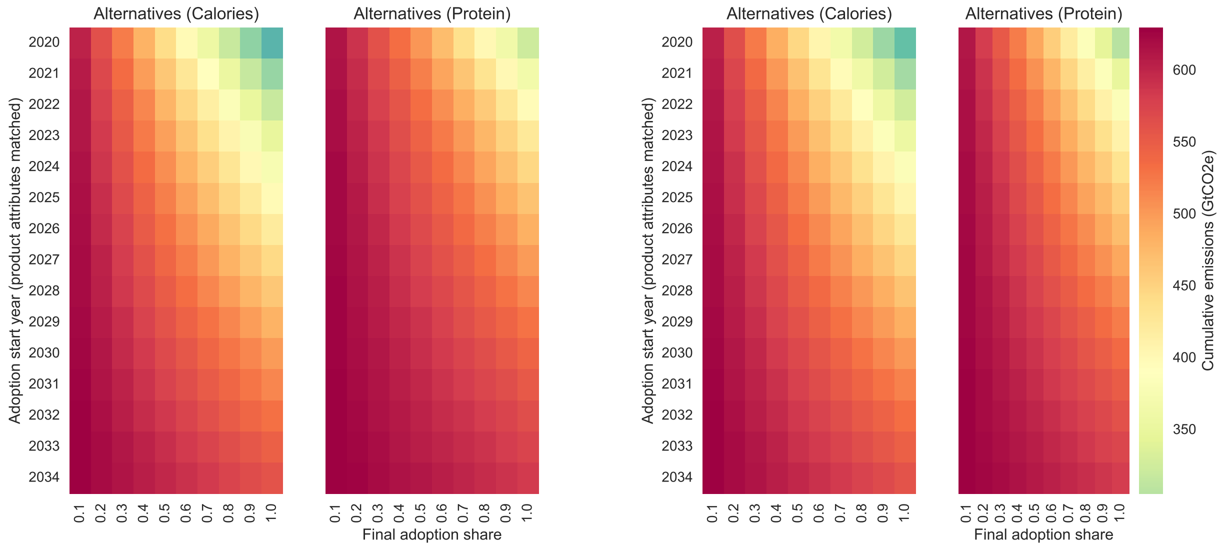

(a) Fast adoption of cultivated

(b) Slow adoption of cultivated

Cumulative emissions are computed for 2020-2050 time period. Alternatives (Calories) is the replacement of ASF with alternatives to match caloric value; Alternatives (Protein) is the replacement of ASF with alternatives to match the total protein demand of the BAU scenario. The adoption start year corresponds to the year in which the attributes of incumbent products are matched by alternatives. The scenario plotted is based on no adoption delay $\delta = 0$ and fast or slow introduction of cultured alternatives to ASF, as indicated. Final adoption share is the 2050 target. The center color of the color bar is set to 390 GtCO2e, estimated emission budget for the food industry and is the same across panels.

References

[1] Bryant, C. & Barnett, J. Consumer Acceptance of Cultured Meat: An Updated Review (2018–2020). *Applied Sciences* **10** (2020). URL https://www.mdpi.com/2076-3417/10/15/5201.

[2] Neuhofer, Z. & Lusk, J. L. Most plant-based meat alternative buyers also buy meat: an analysis of household demographics, habit formation, and buying behavior among meat alternative buyers. *Nature Scientific Report* **12** (2022).

[3] Slade, P. If you build it, will they eat it? Consumer preferences for plant-based and cultured meat burgers. *Appetite* **125**, 428–437 (2018). URL https://www.sciencedirect.com/science/article/pii/S0195666317317531.

[4] Taylor, H., Tonsor, G. T., Lusk, J. L. & Schroeder, T. C. Benchmarking US consumption and perceptions of beef and plant-based proteins. *Applied Economic Perspectives and Policy* **45**, 22–43 (2023). URL https://onlinelibrary.wiley.com/doi/abs/10.1002/aepp.13287. https://onlinelibrary.wiley.com/doi/pdf/10.1002/aepp.13287.

[5] Poore, J. & Nemecek, T. Reducing food's environmental impacts through producers and consumers. *Science* **360**, 987–992 (2018). URL https://www.science.org/doi/abs/10.1126/science.aaq0216. https://www.science.org/doi/pdf/10.1126/science.aaq0216.

[6] Tuomisto, H. L. The eco-friendly burger. *EMBO reports* **20**, e47395 (2019). URL https://www.embopress.org/doi/abs/10.15252/embr.201847395. https://www.embopress.org/doi/pdf/10.15252/embr.201847395.

[7] Mattick, C. S., Landis, A. E., Allenby, B. R. & Genovese, N. J. Anticipatory life cycle analysis of in vitro biomass cultivation for cultured meat production in the united states. *Environmental Science & Technology* **49**, 11941–11949 (2015). PMID: 26383898.

[8] Tuomisto, H. L. & de Mattos, M. J. Environmental impacts of cultured meat production. *Environmental science and technology* **45**, 6117–6123 (2011).
